# Type 1 Interferons and Antiviral CD8 T-Cell Responses

**DOI:** 10.1371/journal.ppat.1002352

**Published:** 2012-01-05

**Authors:** Raymond M. Welsh, Kapil Bahl, Heather D. Marshall, Stina L. Urban

**Affiliations:** Department of Pathology and Program in Immunology and Virology, University of Massachusetts Medical School, Worcester, Massachusetts, United States of America; The Fox Chase Cancer Center, United States of America

Type 1 interferons (IFNs) were the first cytokines discovered and include IFNβ, >ten forms of IFNα, and several other related molecules that all bind to the same type 1 IFN receptor (IFN1R). Type 1 IFNs are commonly referred to as “viral” IFNs because they can be induced directly by virus infections, in contrast to “immune” IFN, or IFNγ, which is synthesized after receptor engagement of T cells and natural killer (NK) cells during immune responses. Type 1 IFNs get induced by viral nucleic acids and proteins acting on cellular signaling molecules such as Toll-like receptors and RNA helicases, which, in turn, release transcription factors into the nucleus. Mice lacking IFN1R appear normal in a pathogen-free environment but are extraordinarily susceptible to virus infections [1]. This susceptibility is partially due to IFN-regulated genes that suppress viral replication, but type 1 IFNs also have many immunoregulatory properties that could also affect host susceptibility to infection.

## 

Indications of the immunoregulatory roles of type 1 IFN came in the 1970s with observations that IFN upregulated the expression of class 1 MHC antigens [2], enhanced histamine secretion by triggered Mast cells [3], and cytolytically activated NK cells [4]–[6]. Several studies showed that addition of IFN to mixed lymphocyte cultures could enhance or inhibit T-cell proliferation, depending on the dose [7]. IFN was then shown to elicit NK cell proliferation in vivo by a mechanism involving the induction of IL-15, a growth factor for NK cells [8], [9]; a similar phenomenon of IFN and IL-15 was later shown for the division of memory T cells [10]. In the past decade a substantial number of new insights have developed in regards to how IFN can directly or indirectly affect T-cell responses to viral infections. IFN can affect T-cell responses by acting on the antigen-presenting cells (APCs), by acting on the T cells, or by inducing other cytokines and chemokines that regulate T-cell responses. Of note is that the phenotype of the T cells and the timing of IFN exposure are of essence, as IFN can inhibit proliferation or induce apoptosis under some circumstances yet be dramatically stimulatory under other conditions. Depending on their activation status, T cells can change their expression levels of IFN1R and their expression of signaling molecules downstream from the IFN1R.

## Mechanisms of IFN Signaling and Gene Activation

All type 1 IFNs bind to a receptor of two chains, IFNαR1, which is constitutively bound to tyrosine kinase 2 (TYK2), and IFNαR2, which is constitutively bound to Janus kinase 1 (JAK1). Ligand binding induces dimerization of both receptor chains and the phosphorylation of TYK2, JAK1, and the intracellular tyrosine residues of each IFN1R chain [11]–[13]. The transphosphorylation of both chains by these kinases results in activation of signal transducers and activators of transcription (STATs) 1 and 2. These form complexes that are translocated into the nucleus and activate the transcription of a wide variety of genes regulated by IFN-stimulated response elements (ISRE) [14], [15]. Type 1 IFNs can limit CD8 T-cell expansion when acting through STAT1, but they can also activate other STATs and promote T-cell expansion when, for example, acting through STAT4 [16], [17]. Type 1 IFNs can also activate STAT 3 and 5, which can mediate antiapoptotic and promitogenic effects in T cells that escape the antimitotic effects of IFN by downregulating STAT1 after activation [13], [18].

**Figure 1:**
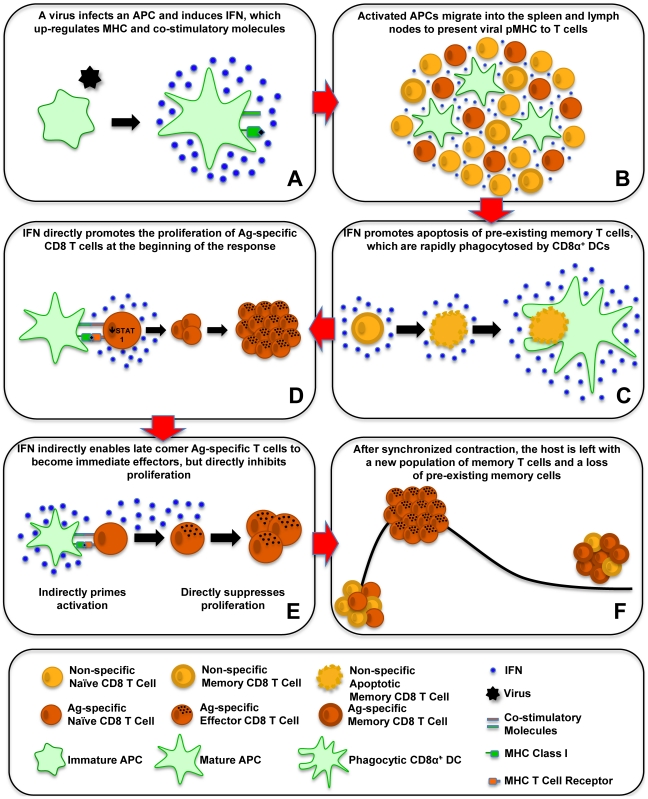
Effect of type 1 IFN on T-cell activation, proliferation, and apoptosis. This schematic shows the effects of type 1 IFN on antiviral CD8 T-cell responses. (A) A virus infects an APC and induces IFN, which upregulates MHC and costimulatory molecules. (B) Activated APCs migrate into the spleen and lymph nodes to present viral pMHC to T cells. (C) IFN promotes apoptosis of preexisting memory T cells, which are rapidly phagocytosed by CD8α+ DCs. (D) IFN directly promotes the proliferation of antigen (Ag)-specific CD8 T cells at the beginning of the response. (E) IFN indirectly enables late comer Ag-specific T cells to become immediate effectors, but directly inhibits proliferation. (F) After synchronized contraction, the host is left with a new population of memory T cells and a loss of preexisting memory cells.

Type 1 IFN plays a major role in the CD8 T-cell response to viral infection, and its effects are on both the APCs (Figure 1A and 1B) and on the T cells (Figure 1D). T cells that are exposed to their cognate peptide antigen presented in the context of MHC (pMHC) on APC-like dendritic cells (DCs) get costimulated through receptors such as CD28 and CD40 ligand and undergo a differentiation program associated with several cycles of division, the expression of the transcription factors t-bet and eomesodermin, followed by the acquisition of effector functions (Figure 1D). These effector functions include cytotoxicity associated with the synthesis of the cytolytic proteins like perforin and the ability to secrete antiviral cytokines such as IFNγ [19]–[22]. Type 1 IFN upregulates expression of both MHC and costimulatory molecules and in so doing can greatly affect the initiation of these T-cell responses (Figure 1A and 1D) [23]. Overall, there is dramatic upregulation of MHC even in nonprofessional APC throughout the host during the course of a viral infection [24].

## Costimulation of CD8 T Cells by Type 1 IFN

Type 1 IFN can provide a major costimulatory effect in its own right by binding to the IFN1R on CD8 T cells and greatly augmenting their proliferation (Figure 1D) [17], [25], [26]. IFNγ, if present, can elicit a similar effect [27]; this was demonstrated in IFN1R bone marrow chimeric mice infected with lymphocytic choriomeningitis virus (LCMV), where the IFN1R+ CD8 cells greatly outgrew the IFN1R- CD8 T cells. Interestingly, this effect was much less profound with vaccinia virus, which is a poor type 1 IFN inducer. Vaccinia virus, however, is a good inducer of IL-12, and IL-12 seems to play a compensatory stimulatory role for T cells in that infection [28]. IFN 1 has potent growth-inhibitory and apoptotic properties, so one might be surprised about this direct augmentation of proliferation. However, as mentioned above, IFN 1–induced growth inhibition is in part mediated through STAT1, but antigen-activated CD8 T cells during LCMV infection downregulate STAT1 and get released from that block [29]. Mice lacking STAT1 experience a putative “nonspecific” proliferation of their CD8 T cells, so it is speculated that IFN 1 signaling through STAT1 may retard nonspecific proliferation and allow the antigen-specific T cells to develop. The action of IFN 1 through other STAT molecules can induce antiapoptotic effects and augment the proliferation of T cells.

## Altered T-Cell Differentiation and Proliferation Caused by Out-of-Sequence Signaling

The timing of IFN exposure can greatly affect the T-cell differentiation pathway and the magnitude of the T-cell response. It is well established that exposure to IFNγ promotes the differentiation of CD4 T cells into IFNγ-secreting Th1 cells [30], [31], but here we are talking about a timing-dependent exposure of CD8 T cells to type 1 IFN. Exposure of naïve CD8 T cells to APC and IFN before exposure to cognate antigen upregulates the T-cell expression of eomesodermin and sensitizes T cells to enter an altered differentiation pathway on encounter with cognate antigen (Figure 1E) [32]. Instead of undergoing several divisions before exerting effector functions, these sensitized CD8 T cells retain a naïve antigenic phenotype but act like memory cells and develop effector-cell properties associated with cytokine production and cytolytic activity within 2–4 h. This is not due to a direct effect of IFN on the T cells, as it occurs even if T cells lack IFN1R. It is more likely due to IFN acting on the APCs, which need to express the restricting MHC molecule for the cognate peptide to sensitize the T cells to respond differently to the cognate peptide.

We propose that the enhanced expression of MHC- presenting self-peptide provides a low level stimulus to naïve T cells, enabling them to retain a naïve T-cell antigenic phenotype yet produce transcription factors that allow them to respond to cognate peptide like a memory T cell.

A common phenomenon occurring during the course of a viral infection is a transient immune deficiency whereby T cells respond poorly to T-cell mitogens in vitro and to challenge with nonviral antigens in vivo [33]; this is, in fact, why one should not get vaccinated during illness. Several phenomena could account for this deficiency, including growth of virus in T cells, impaired antigen presentation, competition for T-cell growth factors, and induction of activation-induced cell death in a Fas ligand-rich environment. However, we have recently shown that type 1 IFN itself may account for much of this immune suppression, if the T cells are exposed to the IFN before cognate antigen encounter (Figure 1E) [34]. Prior exposure to IFN before cognate antigen stimulus impairs the proliferation of T cells after the antigen stimulus, even in the presence of IFN acting as a costimulatory factor, and the inhibition of proliferation in this case requires IFN1R on the T cells. The molecular mechanism for this IFN-induced impairment of proliferation is unknown, but this is reminiscent of earlier work showing that NK cells become hyporesponsive to IFN-mediated activation after having received a prior IFN stimulus [35], [36].

Therefore, T cells that receive an IFN stimulus prior to cognate antigen exposure become sensitized to immediately become effector cells by an indirect IFN-dependent mechanism; but they undergo reduced proliferation by a direct IFN-dependent mechanism. Together these mechanisms may limit de novo T-cell responses in the midst of a viral infection and may aid in the synchronization of the contraction phase of the immune response, because T cells recruited late into the antiviral response would undergo reduced clonal expansion.

## IFN-Induced Apoptosis and Attrition of Memory T Cells

**Figure 2:**
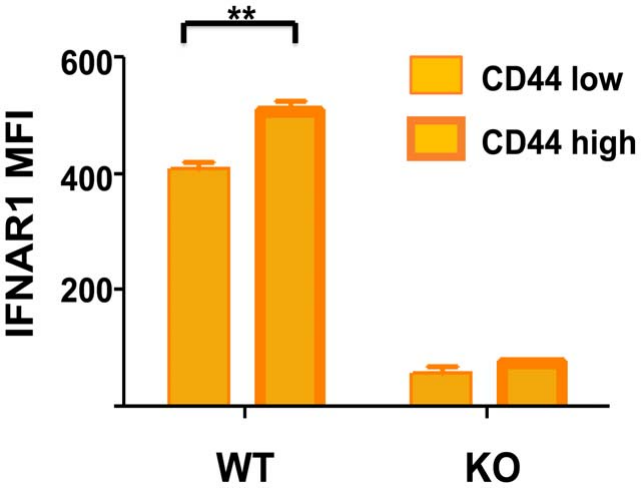
Higher type 1 IFN R (IFNAR1) expression on CD44 high memory phenotype CD8 T cells. Isolated spleen leukocytes from wild-type (WT) or IFNR knockout (KO) mice were stained with fluorescently labeled monoclonal antibodies (mAb) specific for CD8 (53-6.7; BD Pharmingen), CD44 (IM7; BD Pharmingen), and IFNAR-1 (MAR1-5A3; BioLegend). Stained samples were acquired using a BD Biosciences LSR II flow cytometer with FACS Diva software and analyzed with FlowJo software. The mean fluorescence intensity (MFI) for IFNAR1 is shown for CD44 low and CD44 high CD8 T cells, *n* = 3/group. **, *p*<0.005.

IFN-inducing viral infections have a deleterious effect on memory CD8 and CD4 T cells specific to other antigens. We show here that memory-phenotype CD8 T cells express moderately higher levels of IFN1R than do naïve T cells (Figure 2), and it is not unusual for 50%–80% of the memory CD8 T cells to undergo an IFN-induced apoptosis early during infection (Figure 1C) [37]–[40]. Some naïve cells also die in the earlier stages of infection, but to a much lower extent. This apoptosis is associated with elevated caspases, annexin V-staining, and DNA fragmentation and is at least partially dependent on Bim, known to be a proapoptotic molecule induced by type 1 IFN [39], [41]. Of note is that type 1 IFN inducers drive a substantial increase in the number of the highly phagocytic CD8α+, CD11c+ DC population into the spleen of mice (Figure 1B and 1C) [39]. These DC assimilate apoptotic cells and become reactive with Annexin V in the process, making it difficult to quantify apoptotic T cells directly ex vivo and easy to confuse CD8+ T cells with CD8+ DC. The IFN-induced apoptosis of memory T cells can occur in the presence of cognate antigen [38], leading one to question why such a mechanism should exist, as one might want to rapidly recruit antigen-specific memory cells into an immune response. One possibility is that this loss in memory cells is well tolerated because of their initial high frequencies and that it creates room for new T-cell responses to vigorously develop. It has been known for decades that partial depletion of lymphocyte populations can augment new T-cell responses [42], [43]. Further, should these memory T cells cross-react with another pathogen, a reduction in their number may prevent them from overzealously dominating the T-cell response to the cross-reactive epitope [38]. This IFN-induced loss in memory T cells at the beginning of infections would allow for a more diverse and presumably more effective T-cell response to that pathogen. Memory T cells may often be present in clonal excess such that the host can reduce their numbers without deleterious effects. However, a series of infections with heterologous pathogens has been shown to reduce memory T-cell numbers to levels that compromise the host's resistance to infections [44], [45].

## Conclusion: Sequence of Type 1 IFN–Induced Events during a Viral Infection

We now can envisage the series of type 1 IFN–induced events that control CD8 T-cell responses to viral infections (Figure 1). A virus will infect a host and possibly a DC and induce IFN that upregulates MHC and costimulatory molecules, and then the activated DC migrates into the spleen and lymph nodes (Figure 1A and 1B). IFN induces the apoptosis of many of the memory cells and some of the naïve cells, making room in the immune system to drive a strong T-cell response (Figure 1C). The antigen-specific T cells downregulate the antiproliferative STAT1, allowing IFN signals to go through other STAT molecules that inhibit apoptosis and promote proliferation (Figure 1D). Type 1 IFN acts as a strong costimulatory factor driving T-cell expansion. Late comer T cells in the immune response will be indirectly sensitized by IFN to immediately become effector cells but at the expense of proliferation, which is suppressed by direct IFN signaling (Figure 1E). After the virus is cleared, the T-cell response synchronously contracts, leaving the host with a pool of new memory cells and a loss of previously existing ones (Figure 1F).
